# Communication About Sexuality (COSY) Encourages Physical Activity in COPD: A Randomised Trial

**DOI:** 10.2147/COPD.S539514

**Published:** 2025-10-30

**Authors:** Kaba Dalla Lana, Anja Frei, Thomas Radtke, Julia Braun, Milo A Puhan, Claudia Steurer-Stey

**Affiliations:** 1 Epidemiology, Biostatistics and Prevention Institute, University of Zurich, Zurich, Switzerland; 2 mediX Gruppenpraxis Zurich, Zurich, Switzerland

**Keywords:** COPD, quality of life, holistic care, physical activity, sexuality

## Abstract

**Background:**

Communication about sexuality is neglected in chronic obstructive pulmonary disease (COPD). This study evaluated the effectiveness of the Communication about Sexuality intervention (COSY) on quality of life (QoL) and physical activity (PA) and its acceptability in people with COPD.

**Methods:**

People with COPD (GOLD I–IV) were recruited from pulmonary rehabilitation and primary care settings and randomly allocated (1:1 ratio) to the COSY intervention group (IG) or usual care control group (CG). The primary endpoint was change in QoL, assessed with the Control, Autonomy, Self-realization and Pleasure scale (CASP- 12) between baseline and 3 months. The implementation of the intervention was assessed by review of study documents and questionnaires and acceptability by an interview with IG participants at study end.

**Results:**

Thirty-six persons (28% of target sample size), median age 72 years, 44% female, were included and randomized (CG, n=19, IG, n=17). 33 completed the 3-month-follow-up (CG, n=17, IG, n=16). There was no difference in change between the two groups in CASP-12 (mean difference 0.02, 95% CI −2.01 to 2.06). The COSY intervention increased self-efficacy and adherence for PA. All IG participants appreciated the communication about the topic and study participation. Most participants expressed their need for closeness, intimacy and tenderness.

**Conclusion:**

Communication about sexual wellbeing using the COSY instruments was well received by people with COPD and enable healthcare professionals to comfortably address an often-ignored topic. Recruitment challenges limited study power, but the findings offer strong justification for further research into this promising and needed holistic care approach.

## Plain Language Summary

Health care providers and people with chronic obstructive lung disease (COPD) often have trouble talking about intimacy and sex, even though it’s an important part of life and well-being. This study looked at a new approach called the (COmmunication about SexualitY) COSY intervention, which helps healthcare providers talk to patients with COPD about sexuality and emotional needs.

**What was done**:

People with COPD were split into two groups: one got usual care, and the other got the COSY intervention. We wanted to see if COSY improved quality of life and the motivation for regular physical activity. We also wanted to know if patients found COSY helpful and acceptable.

Although the study did not show changes in quality of life after 3 months, people who took part in the COSY programme said these conversations were helpful and meaningful. Many wanted closeness and affection, and the topic of sexual well-being may help motivate them to stay physically active-something that’s very important in COPD care. The COSY approach helped open up important conversations about sexuality that are usually overlooked in COPD care. More research is needed, but this study shows that talking about sexual well-being is both welcomed by patients and useful for more holistic care.

## Introduction

Human sexuality is a fundamental aspect of life and a recognised determinant of quality of life.^1^ Intimacy and an active sex life are linked to mental and physical health benefits.^2–4^ In older adults and those living with chronic illnesses such as COPD, sexual problems and loss of libido are common.^5–9^ Stereotypes—especially those surrounding older adults with chronic conditions—contribute to the widespread neglect of sexuality in relation to wellbeing and quality of life.^2^,^7^

Sexual wellbeing in people with COPD is rarely addressed in clinical care,^6^,^10^,^11^ and remains under-researched. A comprehensive quality of life assessment must include sexual health.^7^,^12^ Reasons for neglect include assumptions that sexuality is irrelevant or inappropriate in older or ill individuals, and that breathlessness during physical and sexual activity renders the topic obsolete.^6^,^8^,^12^ A recent study by Smith et al^13^ found that greater physical activity correlates with better sexual activity in older adults. Improving sexual relationships might on the other hand motivate people with COPD to stay physically active.^14^,^15^ The GOLD 2024 guidelines state: “Patients should be encouraged to increase the level of physical activity although we still don’t know how to best ensure the likelihood of success”.^16^

To help initiate conversations and address this neglected topic, we developed the COSY (COmmunication about SexualitY in COPD) communication instruments,^17^ including material for healthcare professionals and patients. This study aimed to evaluate the effectiveness of the COSY intervention on wellbeing and physical activity in people living with COPD. Additionally, we assessed the acceptability of the intervention to support future implementation.

## Methods

### Study Design

This monocentric randomised controlled trial recruited participants between October 2022 and April 2024. Participants were randomly assigned to the COSY intervention group (IG) or to usual care (control group, CG). The study has been approved by the ethics committee of the canton of Zurich, Switzerland (Kantonale Ethik-Kommission Zürich; BASEC-Nr. 2022–01813 and was registered at ClinicalTrials.gov (NCT05696730). All participants provided written informed consent. The study was performed in line with the principles of the Declaration of Helsinki.

### Participants, Recruitment, Randomisation, and Blinding

Eligibility criteria included age ≥60 years, confirmed COPD diagnosis (post-bronchodilator forced expiratory volume / forced vital capacity ratio<0.7, GOLD I–IV), sufficient German to complete materials, and capacity to provide informed consent. Exclusion criteria were unstable COPD or cardiovascular disease, severe depression, or a life expectancy <1 year.

Participants were recruited via general practitioners, pulmonologists, hospitals, and PR centres in Zurich. Information was provided via flyers, video, and verbal communication. Consenting individuals permitted their contact details to be shared with the study team.

Initial contact was made by phone. The coordinator of the Epidemiology, Biostatistics and Prevention Institute (EBPI) scheduled the baseline assessment (T1) 7–14 days before intervention started. Randomisation (1:1 ratio) was conducted post-T1 using block randomisation stratified by general health status (feeling thermometer: 0–65 vs 66–100). A blinded biostatistician generated the sequence using R; allocation was concealed in Research Electronic Data Capture software (REDCap)^18^ and inaccessible to study staff.

All participants wore a physical activity monitor for seven consecutive days starting at T1. Randomisation results were disclosed after completion of physical activity recording. Due to the nature of the intervention, neither participants nor assessors were blinded, but the data analyst remained blinded to allocation.

### Study Intervention

The COSY intervention comprised a 75-minute face-to-face counselling session followed by two 30-minute telephone calls (weeks 1 and 3). The aim was to sensitise participants to the topic of sexual wellbeing and increase motivation for physical activity by highlighting its relevance to sexual wellbeing.

Counselling followed the COSY instruments.^17^ The COSY instruments consist of four tools: (1) a healthcare professional leaflet, (2) accompanying guidance, (3) a visual spectrum of intimacy, and (4) an illustrated patient booklet. (Supplement 1 overview of the four COSY instruments) These four tools are available free of charge in German, French, Italian and English https://www.lungenliga.ch/fuer-fachpersonen/fachpublikationen-guidelines.

The initial conversation used the COSY leaflet (Supplement 4), which explores four domains of sexual limitation: (1) COPD symptoms, (2) other physical limitations, (3) relationship difficulties, and (4) self-image issues.

**Figure 1:**
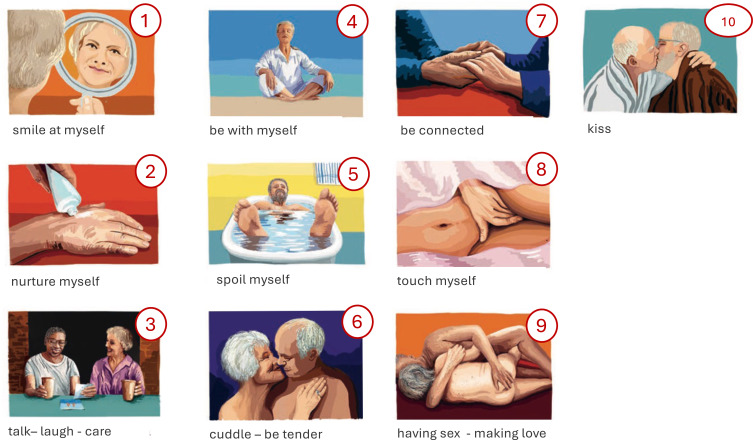
Ten pictograms of possible goals for a fulfilling emotional and intimate life.

During the face-to-face session, the COSY-compass (Supplement 2) was introduced—a supplementary tool to guide goal setting and behaviour change. It includes ten pictograms representing intimacy aspects (Figure 1). The healthcare professional used these to help participants identify a personal goal. Participants then selected motivations, noted challenges, and defined three personalised actions: one for physical activity, one for communication, and one of their choice. For each, self-efficacy was rated (0–10), adherence tracked, and positive outcomes recorded.

Participants received also the COSY patient booklet with six learning targets, pictograms, and suggestions for starting partner communication.

All counselling applied motivational interviewing to promote self-efficacy.^19–21^ The aim was for participants to rate ≥7 in confidence to act.

CG participants received no intervention during the study but were offered a brief session and COSY materials post-T2 (3-month follow-up).

### Outcome Measures

Assessments were conducted at baseline and 3-month follow-up. The primary outcome was change in quality of life in elderly, measured by the CASP-12 German short version,^22^,^23^ covering control, autonomy, self-realisation, and pleasure.

Secondary outcomes included change in physical activity (steps/day, minutes in moderate-to-vigorous physical activity [MVPA]), and perceived activity assessed by the Clinical visit-PROactive Physical Activity instrument (C-PPAC) including the amount of physical activity, difficulty with physical activity and total physical activity experience; scale 0–100).^24^,^25^ Participants wore the triaxial physical activity monitor (ActiGraph wGT3x-BT, Pensacola, FL, USA) around their hips for seven consecutive days after the baseline visit and before the follow-up visit and completed the C-PPAC questionnaire during the visits. The devices were programmed to record raw acceleration with a frequency of 30Hz. Raw data were downloaded and evaluated using the customer software (ActiLife Version 6.13.5) and files with sufficient wear time (ie, at least four days including one weekend day with 10h daily wear time) were exported (60s epoch lengths) and analysed. No filter was used to analyse the data. We used the algorithm by Choi et al^26^ to detect non-wear times, as recommended for this population and the cut-offs from Troiano et al^27^ to calculate time spent sedentary, and in light, moderate, moderate-to-vigorous, and vigorous physical activity. Functional capacity was assessed via one minute sit-to-stand (1-min STS) test.^28^ Additional outcomes included change in Chronic Respiratory Questionnaire (CRQ),^29^ COPD Assessment Test (CAT),^30^ feeling thermometer (FT),^31^ and the Hospital Anxiety and Depression Scale (HADS).^32^ Adverse events and exacerbations were recorded.

Implementation outcomes were derived from COSY-compass data (goals, challenges, self-efficacy, adherence, experiences) and acceptability and experiences by interviews with the participants (Supplement 3) conducted at T2.

### Sample Size and Statistical Analyses

The sample size was based on detecting a 3-point change in CASP-12 (SD = 6.0), representing a moderate effect size (0.5 SD), assuming 80% power, α = 0.05. This required 64 participants per group. Assuming 20% attrition, 160 participants were targeted.

Descriptive statistics are presented as medians (IQR) or counts (percentages). Linear models adjusted for stratification variables were used for group comparisons, for which we show coefficients and 95% confidence intervals. Open-text responses were analysed using conventional content analysis.^33^ All analyses were performed using R version 4.4.2 (R Foundation for Statistical Computing, Vienna, Austria).

## Results

### Study Participants

We contacted 132 healthcare professionals (84 pulmonologists, 48 physiotherapists) to recruit eligible participants. Despite 99% expressing support for the study, only 10% of participants were recruited via these channels.

Most participants (82%) were recruited from the patient pool of the mediX primary care group practice where the study initiators: CS, a pulmonologist, and KD, a respiratory physiotherapist, work. About one in three contacted patients agreed to participate. Reasons for non-participation included health issues (n=24, 28%), ineligibility (n=10, 12%), participation burden (n=9, 11%), other reasons (n=6, 7%) and disinterest in the topic (n=3, 4%). Website and video announcements accounted for 8% of recruited cases.

**Figure 2:**
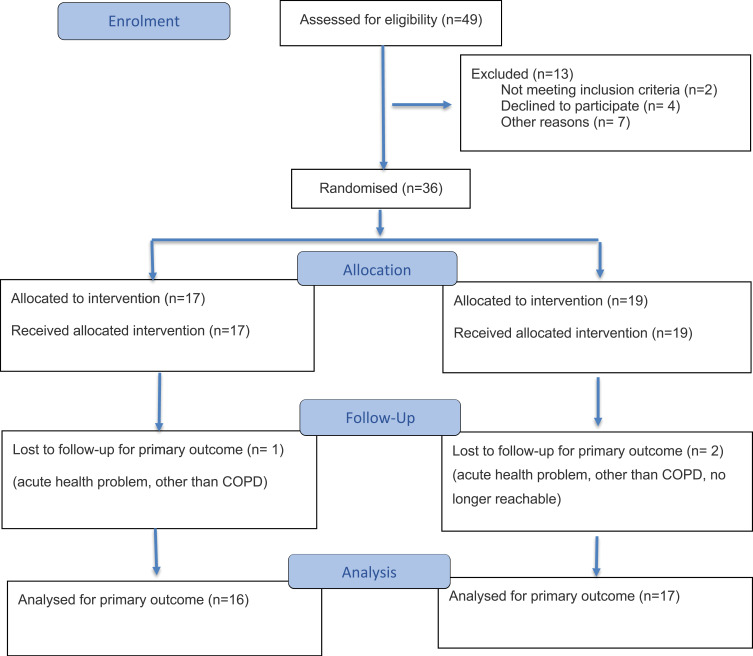
CONSORT flow diagram of the progress through the phases of the randomised trial.

In total, 49 individuals were screened between October 2022 and April 2024. Thirty-six participants (median age: 72.5 years; 44% female; 56% partnered; 86% former or current smokers) were included and randomised (19 CG, 17 IG). Thirty-three completed the 3-month follow-up (17 CG, 16 IG). See Table 1 for demographics and Figure 2 for patient flow. Based on the information in Table 1, the participants in the IG were a little younger with a slightly higher proportion of smokers and fewer exacerbations in the previous year compared to the CG.

**Table 1:** Baseline Demographics

	Overall (n=36)	Intervention (n=17)	Control (n=19)
Age (years)	72.50[65.75, 75.25]	67.00[64.00, 74.00]	74.00[70.50, 78.50]
Female sex, n (%)	16 (44.4)	8 (47.1)	8 (42.1)
Living in partnership, n (%)	25 (69.4)	12 (70.6)	13 (68.4)
FEV_1_ (L)	1.48 [1.09, 1.77]	1.38 [1.02, 1.72]	1.50 [1.13, 1.78]
FEV_1_ (%predicted)	62.1 [45.8, 75.1]	57.7 [37.2, 69.6]	62.4 [47.1, 83.8]
FEV_1_ (z-score)	−2.29 [−3.07, −1.58]	−2.45 [−3.83, −1.75]	−2.21 [−2.82, −0.96]
FEV_1_/FVC ratio	0.59 [0.53, 0.65]	0.62 [0.52, 0.67]	0.58 [0.54, 0.65]
GOLD stages, n (%)			
GOLD 1	8 (22.2)	3 (17.6)	5 (26.3)
GOLD 2	15 (41.6)	7 (41.2)	8 (42.1)
GOLD 3	9 (25.0)	4 (23.5)	5 (26.3)
GOLD 4	4 (11.1)	3 (17.6)	1(5.3)
Current-smoker, n (%)	8 (22.2)	5 (29.4)	3 (15.8)
Ex-smoker, n (%)	23 (63.9)	10 (58.8)	13 (68.4)
Never-smoker, n (%)	5 (13.9)	2 (11.8)	3 (15.8)
Number of comorbidities	3 [2, 4]	3 [2, 4]	3 [2, 4]
Exacerbations previous 12 months (at least 1) n (%)	17 (47.2)	7 (41.2)	10 (52.6)
Feeling thermometer >65, n (%)	24 (66.7)	11 (64.7)	13 (68.4)

**Notes**: Data are n (%) or median [interquartile range].

**Abbreviations**: FEV_1,_ Forced expiratory volume in first second; FVC, Forced vital capacity; GOLD, Global initiative for Obstructive Lung Disease.

### Effectiveness

There was no statistically significant between-group difference in CASP-12 total score change after 3 months (adjusted coefficient: 0.02, 95% CI –2.01 to 2.06). Similarly, no significant differences were observed in the change of CRQ or CAT scores.

No between-group differences emerged in change in daily steps, MVPA, or C-PPAC domains. A between-group difference was noted for the change of the 1-min STS test in favour of the IG (4.11 repetitions; 95% CI −0.68 to 8.9) (Table 2).

**Table 2:** Descriptives of Primary and Secondary Outcomes at Baseline and at 3 months in the Intervention and Control Groups Along with Coefficients for the Difference in Change Over Time Between the Two Groups From Linear Models, Adjusted for Feeling Thermometer

Primary endpoint	Intervention		Control		Adjusted Comparison of Change Between Groups	
	Baseline (n=17)	3 Months (n=16)	Baseline (n=19)	3 Months (n=17)	Coefficient (95% CI)	p-value
	Median (IQR)	Median (IQR)	Median (IQR)	Median (IQR)		
Quality of life CASP-12	26.00 [25.00, 27.00]	26.00 [25.00, 28.00]	25.00 [24.00, 27.00]	26.00 [25.00, 28.00]	0.02 [−2.01 to 2.06]	0.98
**Secondary endpoints**						
C-PPAC difficulty score	77.00 [56.00, 89.00]	79.00 [63.00, 85.25]	81.00 [67.00, 91.50]	83.00 [72.00, 86.00]	2.97 [−3.18 to 9.12]	0.33
C-PPAC amount score	72.50 [58.75, 81.00]	70.00 [65.00, 78.00]	70.00 [62.00, 75.00]	65.00 [57.00, 75.00]	2.05 [−4.09 to 8.19]	0.50
C-PPAC total score	74.50 [61.12, 83.50]	74.00 [63.25, 83.75]	77.25 [70.75, 80.12]	76.50 [66.00, 80.00]	2.46 [−1.39 to 6.31]	0.20
Number of steps /day	5604.50 [3442.75, 8045.00]	6068.00 [4159.50, 6664.50]	4081.50 [3314.50, 7321.00]	4897.00 [3109.50,6469.00]	945.87 [−775.17 to 2666.91]	0.27
MVPA, min per day	13.00 [4.18, 25.15]	17.60 [5.85, 27.55]	9.70 [4.40, 39.90]	10.20 [2.70, 38.65]	8.9 (−7.89 to 25.69)	0.29
1-min STS test, repetitions	32.00 [27.75, 40.00]	34.00 [30.00, 47.50]	29 (25.00,41.50)	29 [24.00, 41.00]	4.11 (−0.68 to 8.9)	0.09
CRQ Dyspnea, scale 1-7	5.20 [3.60, 6.40]	5.30 [4.40, 6.45]	5.60 (4.15,6.55)	5.80 [4.80, 6.40]	0.36 (−0.27 to 0.99)	0.25
CRQ Fatigue, scale 1-7	5.00 [2.75, 5.50]	5.00 [3.94, 5.75]	5.25 (4.38,5.75)	5.50 [4.50, 6.25]	0.11 (−0.41 to 0.63)	0.67
CRQ Emotional function, scale 1-7	5.00 [4.57, 5.57]	5.14 [4.71, 5.71]	5.29 (4.79,6.14)	5.71 [5.14, 6.29]	0 (−0.46 to 0.46)	1.00
CRQ Mastery, scale 1-7	5.00 [4.75, 6.50]	5.50 [5.19, 6.50]	6.75 (5.25,7.00)	6.75 [5.75, 6.75]	0.06 (−0.35 to 0.47)	0.77
CAT score, scale 0-5	13.00 [7.00, 19.00]	12.50 [9.25, 17.00]	12.00 (4.00,13.50)	10.00 [5.00, 19.00]	−1.84 (−5.28 to 1.59)	0.28
HADS depression, scale 0-21	6.00 (5.00,9.00)	6.00 [3.75, 6.50]	5.00 (3.25,5.75)	5.00 [4.00, 6.00]	−0.86 (−2.17 to 0.46)	0.19
HADS anxiety	5.00 [3.00, 8.00]	4.00 [1.75, 6.00]	4.00 [2.00, 6.50]	2.00 [1.00, 6.00]	−1.03 (−2.41 to 0.35)	0.14
FT (median [IQR])	80.00 [60.00, 90.00]	80.00 [67.50, 86.25]	75.00 [65.00, 81.50]	80.00 [60.00, 85.00]	1.61 (−9. 28 to 12.50)	0.77

**Abbreviations**: CASP-12 Control, Autonomy, Self-realisation and Pleasure German short-version questionnaire; C-PPAC, Clinical visit-PROactive Physical Activity; MVPA, Moderate-to-vigorous physical activity; CRQ, Chronic Respiratory Questionnaire; STS, Sit To Stand; CAT COPD Assessment Test; HADS, Hospital Anxiety and Depression Scale, FT, Feeling thermometer.

### Acceptability of the Intervention

#### Patient-Reported Limitations in Sexual Wellbeing

Seventeen COSY communication leaflets were analysed. COPD symptoms (Level 1) were the leading limitation for 18% of participants (mean severity 7/10). All these participants aimed to maintain or enhance sexual activity by improving fitness and reducing dyspnoea.

Other physical limitations (Level 2) were mentioned by 29% (eg, erectile dysfunction, back pain). Relationship difficulties (Level 3) were cited by three patients. Self-image issues (Level 4), such as body weight, were reported by one participant. Desired goals included maintaining (21%), rediscovering (21%), or intensifying (36%) sexual activity; 29% reported no current limitations.

### COSY-Compass Insights

#### Intimacy Goals and Motivations

Sixteen COSY compasses were returned and analysed.

From the ten pictograms representing intimacy, the most commonly selected was “talk-laugh-care” (n=9), followed by “cuddle-be tender” (n=3), “sex-making love” (n=2), and others. Motivations included “being accepted for who I am” (n=6), “more physical closeness” (n=2), “enjoyment in intimate life” (n=1), and “less breathlessness” (n=2). No participants selected “more safety and control”.

### Actions and Adherence

All IG participants selected individualised physical activity goals. Recorded adherence over the 91-day period ranged from 0 to 96 activities (mean 51). Reported positive experiences ranged from 0 to 5 (mean 1.6 per person).

Communication goals were chosen by 73% of participants. Recorded communication activities ranged from 1 to 53 (mean 11), with a similar mean of 1.6 positive experiences.

Self-selected actions included secretion mobilisation (n=2) and relaxation techniques (n=3).

### Self-Efficacy

Average self-efficacy for physical activity increased from 8.2 (week 1) to 8.9 (week 3) and 9.0 (study end). Common plans included gym visits, walks, home training (eg, myhomex.ch), cycling instead of driving, and walking to work.

Average self-efficacy for communication decreased from 8.3 to 6.5. For other self-selected actions, initial efficacy averaged 8.4, slightly declined to 7.2 by study end.

### Participant Feedback

Sixteen of 17 IG participants reported enjoying the intervention. All valued the opportunity to discuss closeness, intimacy, and tenderness. The face-to-face conversation was considered the most motivating component (88%). Patients appreciated learning about the relationship between physical activity, sexual wellbeing, and physical activity and COPD prognosis. COSY tools were viewed as helpful reminders. Criticisms focused mainly on the extent of documentation and pre-inclusion information.

## Discussion

The COSY intervention was highly appreciated by participants, who welcomed the opportunity to discuss sexual wellbeing in a respectful and supportive setting. No significant differences were observed in change of Qol but COSY enhanced self-efficacy and adherence to individualised physical activity plans. Given that sexuality is a fundamental aspect of life and a recognised determinant of Qol it can be assumed that an effect on QoL takes time and that the duration of the study was correspondingly too short.

Hinchliff et al^34^ emphasise the “Three Ps” of raising sexual issues in clinical practice: privacy, permission, and practice. COSY’s multimodal and partly non-verbal tools enabled participants to articulate their desires and needs related to intimacy and tenderness.

Despite strong support from clinicians, recruitment challenges revealed a disconnect between professional attitudes and actual practice. Health professionals often report discomfort or uncertainty when addressing sexual health in elderly patients with chronic diseases,^35^ and may prioritise other clinical issues.^7^,^12^ These barriers also affected our study. Nevertheless, our experience shows that recruitment is feasible when the topic is addressed confidently and empathetically. One in three contacted patients agreed to participate—likely due to the personal openness of the study staff and their ability to introduce the topic appropriately. Among those who were invited but did not participate, the most commonly reported reason was the presence of other health issues. Depending on social norms, patients may also feel psychologically burdened by concerns about sexual communication and reluctant to disclose these to medical professionals. However, disinterest with the topic was the least mentioned reason for non-participation in our study. Previous research has shown that older patients want their healthcare providers to ask about sexual issues,^36^ and that it is easier for patients when clinicians initiate the conversation. The COSY communication instruments may support healthcare providers in “breaking the ice”.

In our study, 18% of participants cited COPD as the main cause of reduced sexual wellbeing. A systematic review^37^ found even higher prevalence (48–82%), and a clear association between sexual limitations and reduced quality of life. Sexual activity and intimacy are known to support mental and physical wellbeing and coping with chronic conditions.^35^

COSY linked quality of life to regular physical activity and sexual wellbeing. Smith et al^13^ similarly found associations between moderate physical activity and increased sexual activity generally in older people. These connections were actively communicated during the intervention. Although no statistically significant improvement was found in the primary outcome (QoL) or COPD-specific QoL measures,^29^ COSY enhanced self-efficacy and adherence to individualised physical activity plans. These changes were reflected in trends toward more physical activity and improvements in the 1-min STS test in the IG.

### Strengths and Limitations

A major strength of this study lies in its focus on a largely neglected aspect of COPD care. While we could not show an effect for the primary outcome, qualitative and behavioural data highlight that sexual wellbeing is both relevant and desired by people living with COPD. Furthermore, it may serve as an intrinsic motivator to sustain physical activity—one of the most pressing challenges in pulmonary rehabilitation.

The main limitation is the underpowered sample, which prevents definitive conclusions about intervention efficacy.

### Implications for Practice and Research

Our findings reveal unmet needs for communication about sexuality in COPD as part of holistic, patient-centred care. A culture of openness is needed. COSY tools offer practical guidance to healthcare professionals, making it easier to raise the topic in a structured and sensitive manner. Addressing sexual wellbeing alongside physical activity in PR and routine care may help avoid stereotypes and stigma.

Future research should test the COSY intervention in larger samples and over longer follow-up periods to assess long-term effects on quality of life and physical activity behaviour.

## Conclusion

Communication about sexual wellbeing using the COSY instruments were well received by people with COPD and can enable healthcare professionals to comfortably address an often-ignored topic. While no significant between group differences were observed in quality of life, the intervention improved self-efficacy and patient engagement. Recruitment challenges limited study power, but the findings offer strong justification for further research into this promising and needed approach.

## Data Availability

The authors confirm that the data supporting the findings of this study are available within the article and /or its Supplementary Materials. Deidentified participant data will be made available upon request from the corresponding author CS. Data transfers will need to comply with the data transfer agreement. COSY instruments are available for free https://www.lungenliga.ch/fuer-fachpersonen/fachpublikationen-guidelines.
